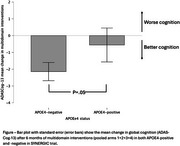# Does *APOE4* genotype moderate the effects of multidomain exercise interventions in older individuals living with mild cognitive impairment?

**DOI:** 10.1002/alz70860_103664

**Published:** 2025-12-23

**Authors:** Frederico Pieruccini‐Faria, Surim Son, Teresa Liu‐Ambrose, Amer M. Burhan, Quincy J Almeida, Laura E. Middleton, Karen Li, Sarah Fraser, Louis Bherer, Manuel Montero‐Odasso

**Affiliations:** ^1^ Gait and Brain Laboratory, Parkwood Institute, London, ON, Canada; ^2^ Gait & Brain Lab; Lawson Research Institute; Schulich School of Medicine& Dentistry, Division of Geriatric Medicine, Western University, London, ON, Canada; ^3^ Schulich School of Medicine & Dentistry, Western University, London, ON, Canada; ^4^ Parkwood Institute, London, ON, Canada; ^5^ Vancouver Coastal Health Research Institute, Vancouver, BC, Canada; ^6^ Centre for Aging SMART, Vancouver Coastal Health Research Institute, Vancouver, BC, Canada; ^7^ University of British Columbia, Vancouver, BC, Canada; ^8^ Djavad Mowafaghian Centre for Brain Health, Vancouver, BC, Canada; ^9^ University of Toronto, Toronto, ON, Canada; ^10^ Care Space Health, Waterloo, ON, Canada; ^11^ Schlegel‐UW Research Institute for Aging, Waterloo, ON, Canada; ^12^ University of Waterloo, Waterloo, ON, Canada; ^13^ Concordia University, Montreal, QC, Canada; ^14^ University of Ottawa, Ottawa, ON, Canada; ^15^ Centre de Recherche de l'Institut Universitaire de Gériatrie de Montréal, Montréal, QC, Canada; ^16^ University of Montreal, Montreal, QC, Canada; ^17^ Schulich School of Medicine & Dentistry, Division of Geriatric Medicine, Western University, London, ON, Canada

## Abstract

**Background:**

Mild Cognitive Impairment (MCI) may be associated with the presence of the apolipoprotein E4 allele (APOE4), a genetic factor linked to accelerated cognitive decline. The SYNERGIC trial demonstrated that physical exercise (PE) involving aerobic and resistance training improves global cognition in individuals with MCI, with significantly enhanced benefits of cognitive training but not vitamin D. The role of APOE4 status in responsiveness to these interventions remains unclear.

**Method:**

Of 175 participants in the SYNERGIC trial, 80 underwent APOE4 genotyping. This study analyzed 67 participants (mean age: 73.8 years; 43.2% women) who completed a 6‐month multidomain intervention across four arms: (1) PE + Cognitive training (Neuropeak®) + Vitamin D; (2) PE + Cognitive training; (3) PE + Vitamin D; and (4) PE alone. Changes in global cognition (ADAS‐Cog‐13) were compared between APOE4‐positive and APOE4‐negative individuals using ANOVA.

**Result:**

APOE4‐positive participants showed attenuated global cognitive improvements from multidomain interventions compared to APOE4‐negative participants (mean difference = ‐2.08, 95% CI: ‐4.18 to 0.11; *p* = 0.052; d = ‐0.56). A significant group effect (*p* = 0.03) revealed that the PE + Vitamin D arm worsened global cognition over time compared to all other arms, with significant decline relative to the arm receiving all three interventions (*p* = 0.02). Sensitivity analysis excluding arms with vitamin D confirmed the attenuated effect of APOE4‐positive status on global cognition (mean difference = ‐2.99, 95% CI: ‐6.10 to 0.11; *p* = 0.05; d = ‐0.78).

**Conclusion:**

Individuals with MCI and APOE4‐positive status exhibit reduced responsiveness to multidomain interventions for global cognition, likely due to underlying genetic neurodegeneration.